# Supplementary figures and images for: A mixed methods study to evaluate participatory mapping for rural water safety planning in western Kenya
Source: PLoS One. 2021 Jul 28;16(7):e0255286. doi: 10.1371/journal.pone.0255286 (PMC8318241; doi:10.1371/journal.pone.0255286)

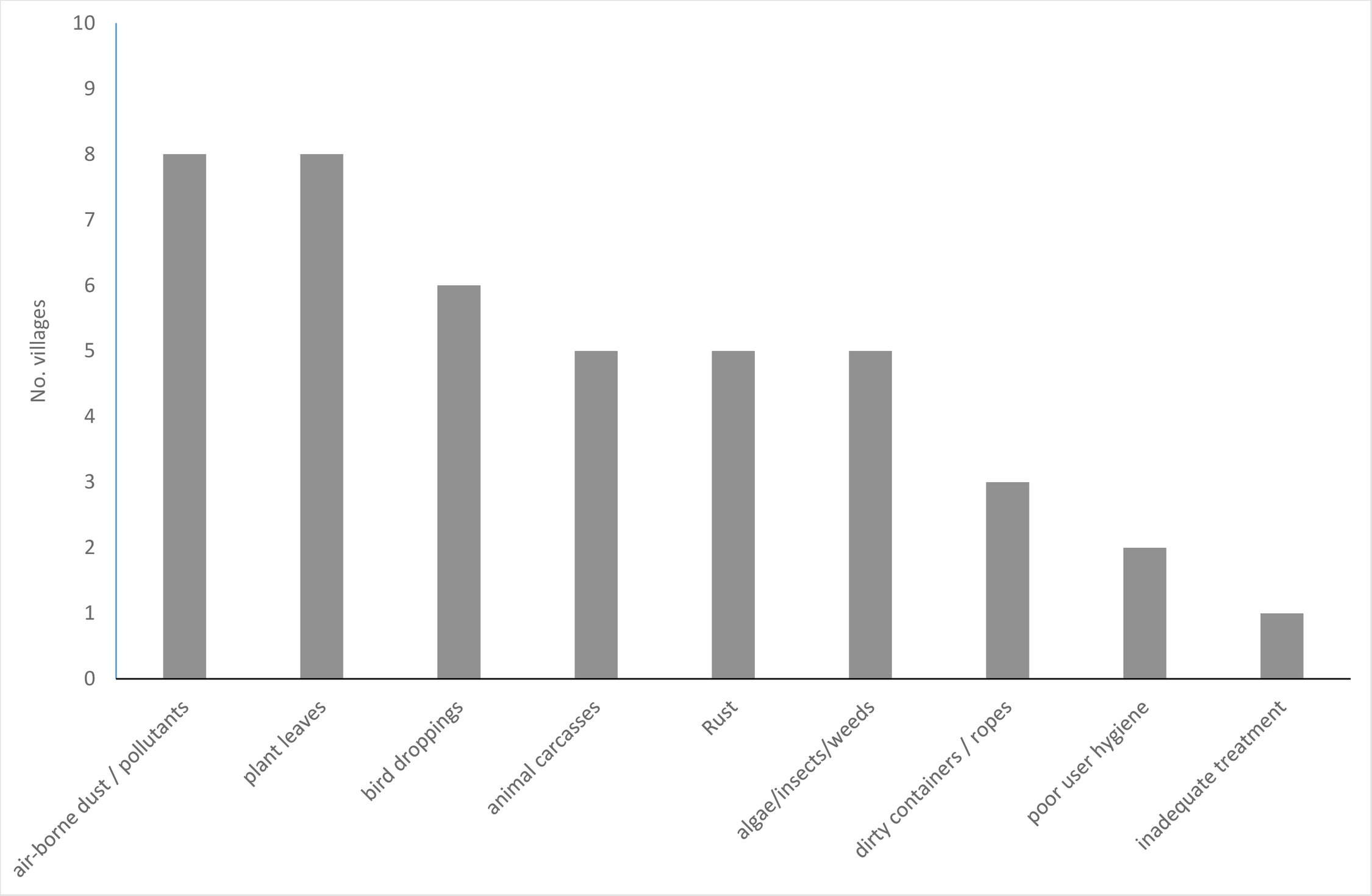

Supplement: S1 Fig — (TIF) [file pone.0255286.s001.tif]

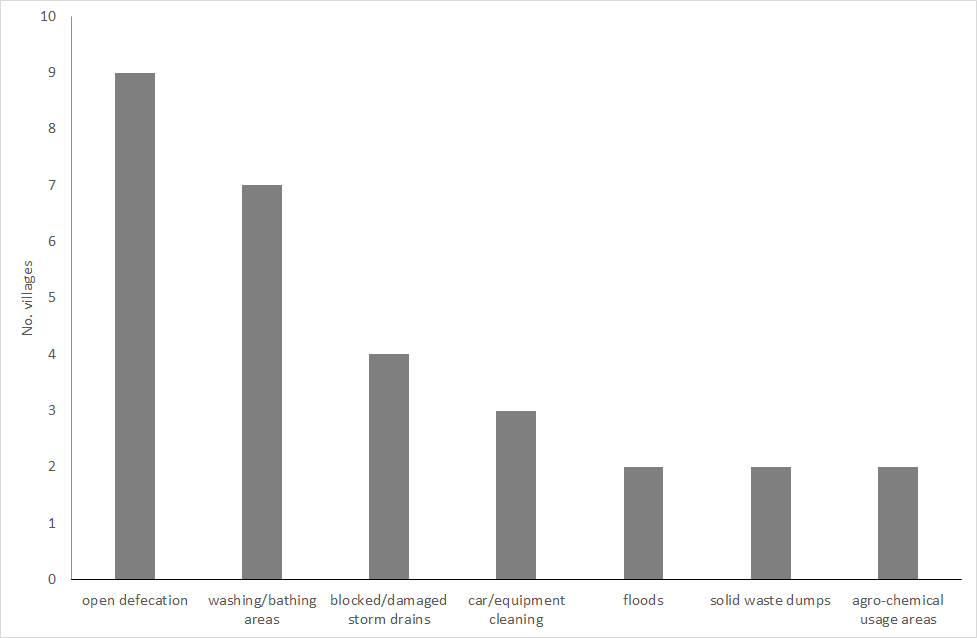

Supplement: S2 Fig — (TIF) [file pone.0255286.s002.tif]

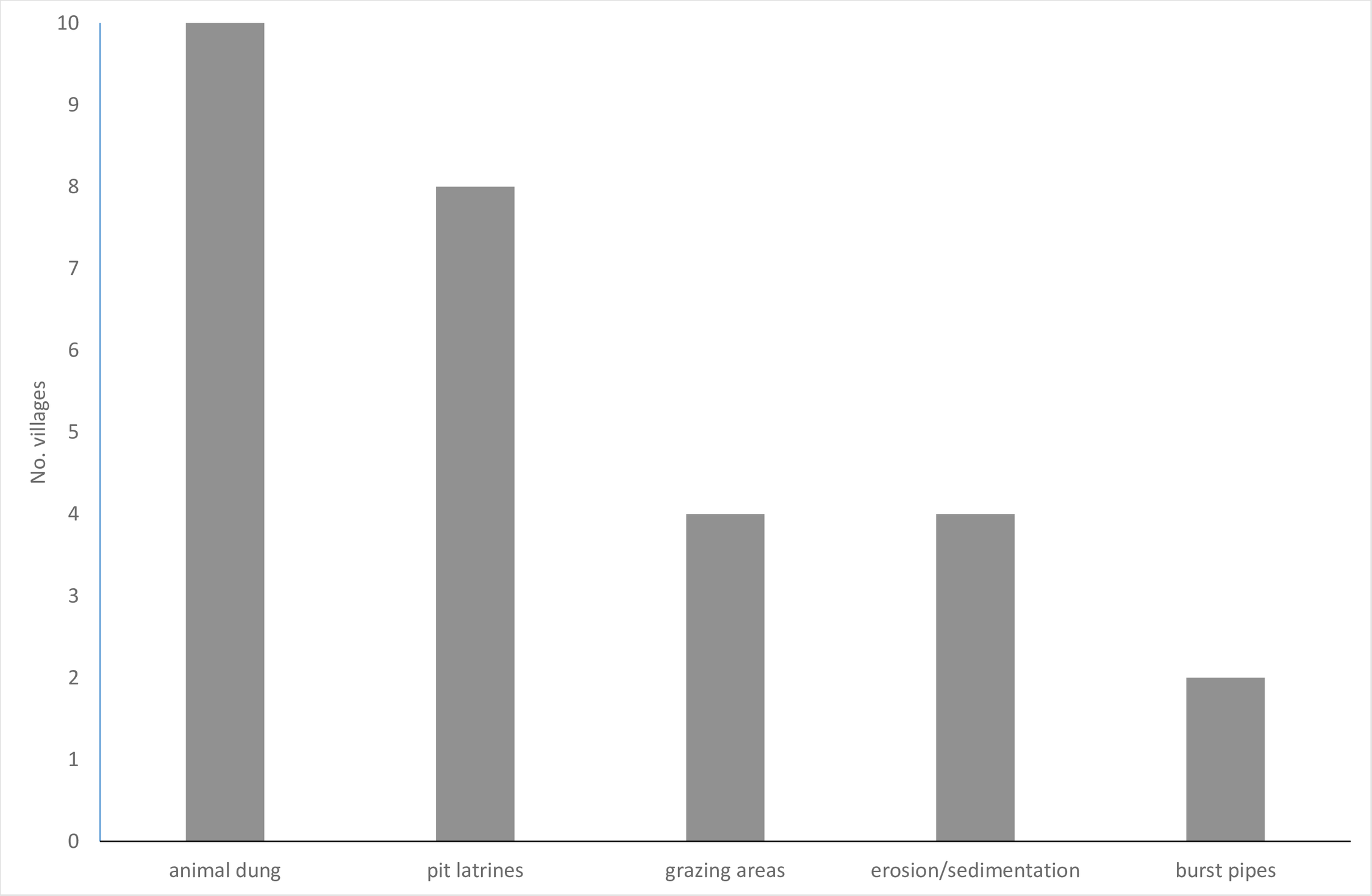

Supplement: S3 Fig — (TIF) [file pone.0255286.s003.tif]
