# Supplementary material for: A mixed methods study to evaluate participatory mapping for rural water safety planning in western Kenya
Source: PLoS One. 2021 Jul 28;16(7):e0255286. doi: 10.1371/journal.pone.0255286 (PMC8318241; doi:10.1371/journal.pone.0255286)
Supplement: S2 Table — (DOCX) [file pone.0255286.s005.docx]

**Article Title:** A mixed methods study to evaluate participatory mapping for rural water safety planning in western Kenya

**Journal name**: PLoS ONE

**Names of the authors:**

Joseph Okotto-Okotto, Weiyu Yu, Emmah Kwoba, SM Thumbi, Lorna G. Okotto, Peggy Wanza, Diogo Trajano Gomes da Silva, Jim A. Wright*

*** Corresponding author**: School of Geography and Environmental Science, University of Southampton, UK. Email: [j.a.wright@soton.ac.uk](mailto:j.a.wright@soton.ac.uk)

**S2 Table. Table showing physico-chemical and organoleptic water properties from a water source survey in ten villages in Siaya County**

| Source type | No. samples | Median turbidity (NTU*, 5^th^ – 95^th^ centile) | Median electro-conductivity (μS; 5^th^ – 95^th^ centile) | % visibly coloured, cloudy or with particles |
| --- | --- | --- | --- | --- |
| Improved sources: Piped/kiosk | 43 | 3.8 (0.0 - 30.5) | 182.0 (0.0 - 323.4) | 0.0% |
| Borehole | 6 | 1.2 (0.1 - 41.8) | 1062.0 (323.0 - 3015.0) | 0.0% |
| Rainwater | 46 | 2.0 (0.2 - 9.3) | 30.0 (9.0 - 172.9) | 2.2% |
| Predominantly unimproved sources:  Well | 23 | 6.4 (0.2 - 318.2) | 518.0 (133.2 - 2398.4) | 30.4% |
| Spring | 5 | 114.0 (40.8 - 295.0) | 245.0 (131.0 - 833.0) | 100.0% |
| Surface water | 45 | 144.0 (7.1 - 1000.0) | 190.0 (43.2 - 1542.1) | 66.7% |
| Unclassified sources:  Rainwater-piped hybrid | 6 | 2.7 (0.0 - 28.9) | 151.5 (14.0 - 1031.0) | 16.7% |

* NTU: Nephelometric Turbidity Units
