# Supplementary material for: A mixed methods study to evaluate participatory mapping for rural water safety planning in western Kenya
Source: PLoS One. 2021 Jul 28;16(7):e0255286. doi: 10.1371/journal.pone.0255286 (PMC8318241; doi:10.1371/journal.pone.0255286)
Supplement: S3 Table — (DOCX) [file pone.0255286.s006.docx]

**Article Title:** A mixed methods study to evaluate participatory mapping for rural water safety planning in western Kenya

**Journal name**: PLoS ONE

**Names of the authors:**

Joseph Okotto-Okotto, Weiyu Yu, Emmah Kwoba, SM Thumbi, Lorna G. Okotto, Peggy Wanza, Diogo Trajano Gomes da Silva, Jim A. Wright*

*** Corresponding author**: School of Geography and Environmental Science, University of Southampton, UK. Email: [j.a.wright@soton.ac.uk](mailto:j.a.wright@soton.ac.uk)

**S3 Table.** **Rankings of community interventions proposed by participants during follow-up meetings.**

| **COMMUNITY PROPOSALS** | **VILLAGE NAME** | | | | | | | | | | **MEDIAN RANK** |
| --- | --- | --- | --- | --- | --- | --- | --- | --- | --- | --- | --- |
|  | **Ndwara** | **Ongielo** | **Kamin**  **ogedo** | **Lwak** | **Sinogo** | **Got Bondo** | **Rambugu** | **Sangla** | **Siger** | **Wang’arot** |  |
| Well remediation | 2 | - | 2 | 2 | - | 2 | - | 2 | 3 | - | 2 |
| Structures separating livestock and people at water pans | - | 3 | 3 | 3 | 2 | - | - | 3 | 4 | 2 | 3 |
| Chlorine dispenser installation at water points | 3 | 2 | 5 | 4 | 6 | 6 | 2 | 4 | - | 3 | 4 |
| Construction of River/Spring protection including catchment conservation | 5 | 5 | 4 | 6 | 3 | 3 | 4 | 7 | - | - | 4.5 |
| Borehole construction* | 6 | - | - | - | - |  | 3 |  | 2 |  | 3 |
| Installation / remediation of rainwater harvesting infrastructure* | 1 | 1 | 1 | 1 | 1 | 1 | 1 | 1 | 1 | 1 | 1 |
| Community mobilization, educational and hygiene awareness programmes (e.g. household water treatment) * | 7 | 4 | 6 | 5 | 5 | 4 | 5 | 5 | 5 | 4 | 5 |
| Woodlot development for firewood to boil water* | 4 | 6 | 5 | 7 | 4 | 5 | 6 | 5 | 6 | 5 | 5 |
| - source intervention not ranked because source not available in village  * intervention implementable by community with minimal external support | | | | | | | | | | | |
