# Supplementary material for: A mixed methods study to evaluate participatory mapping for rural water safety planning in western Kenya
Source: PLoS One. 2021 Jul 28;16(7):e0255286. doi: 10.1371/journal.pone.0255286 (PMC8318241; doi:10.1371/journal.pone.0255286)
Supplement: S1 Protocol — (DOCX) [file pone.0255286.s007.docx]

**Article Title:** A mixed methods study to evaluate participatory mapping for rural water safety planning in western Kenya

**Journal name**: PLoS ONE

**Names of the authors:**

Joseph Okotto-Okotto, Weiyu Yu, Emmah Kwoba, SM Thumbi, Lorna G. Okotto, Peggy Wanza, Diogo Trajano Gomes da Silva, Jim A. Wright*

*** Corresponding author**: School of Geography and Environmental Science, University of Southampton, UK. Email: [j.a.wright@soton.ac.uk](mailto:j.a.wright@soton.ac.uk)

**S1 Protocol. Household Survey Questionnaire**

Interviewer ID code: ___________________ Date: __ __ / __ __ / __ __ *(dd/mm/yy)*

*Namba mar Yango Japenjo Tarik*

##

##

Village ID code: _______________ Village name: _______________

*Namab mar Yango gweng’ Nying’ gweng’*

Compound ID: ___/___ /___ Household ID: ___/___ /___

*Yango dala*  *Yango ot*

Name of youngest child-------------------(Select from drop down list)

### **HOUSEHOLD CONSENTING AND ELIGIBILITY**

**Q1. Is the Household Eligible? ________**

YES =>

NO

**Q1a. If NO – Reason for ineligibility ________**

No adult occupier (above 18years)

No child under 5 years

No longer in IEIP

Withdrawal

Other reason – specify _____________

**(End of questionnaire if household is ineligible)**

**Q1b. Respondent’s details**

**Respondent’s date of birth**? -----------------------(select year)

**If the respondent is below 18 years of age, probe if:**

Married

Respondent is the household head

Respondent is pregnant/with children

**Respondent’s age__ (auto fill)**

**Respondent’s gender**

Male

Female

**Is the respondent the Household head?**

Yes

No

**if yes, Skip to Q1d**

**Q1c.** **What is the relationship of the respondent to the household head? ________** **(Spouse/Son/Daughter/Brother/Sister/Uncle/Aunt/Nephew/Niece/Grandchild/Other)** *Note: respondents should be aged 18 years or over. Do not ask those younger than this age to respond to the survey.*

If “Other” – Specify

**Q1d. Do you have a Mobile Phone?**

YES/No)

**Mobile phone number** ____________

**Q2a. What are the water sources your household uses for drinking during the dry season?**

W1. Piped water – into dwelling/*Pi odundu (pep) Ma odonjo nyaka kar jot*

W2. piped to yard or plot/*Pi odundu (pep) mantie e plot*

W3. public tap or standpipe/*Freji mar ji duto kata odundu (pep) mochung mar pi,*

W4. tubewell or borehole/*Bur mar pi matut mokuny gi masin (kisima)*

w5. - protected well /*Bur mar pi ma Okuny ma tut ma oriti’*

w6. – unprotected well / *Bur mar pi ma okuny ma tut ma ok oriti’*

W7. – protected spring/ *Soko mar pi moriti*

W7A. – unprotected spring/*Soko mar pi ma ok oriti* *(i.e. springs without protection like box or concrete walls shown in protected spring pictures) Kuom ranyisi, ma onge kod gik ma oritgi godo kaka boxi kata kor ma omuon kod kokoto gi simiti ma oket kaka ranyisi e pichni go.*

W8. rainwater /*Pi Koth*

w9. bottled water/*Pi Chupa*

w11. tanker truck /*Tange mar Lori*

w10. cart with small tank/ *Tange matin mar kanyna*

w12a– free-flowing river or stream/*Pi Aora Mamol*

W12B-stagnant water in dam, pond or lake/*Pi mochung’ mar yawo, dago kata nam*

W12C– canal or irrigation channel/ *Pi mar riwa/oula kata riwa mag miyo ni cham pi (rigesen)*

other: specifY _____________ *Machielo –wache adimba*

**Q2aR1**. **Source of piped water during the dry season.**

Utility

Borehole

Lakewater system

Don’t know

**Q2b. What are the water sources your household uses for drinking during the wet season?**

W1. Piped water – into dwelling/*Pi odundu (pep) Ma odonjo nyaka kar jot*

W2. piped to yard or plot/*Pi odundu (pep) mantie e plot*

W3. public tap or standpipe/*Freji mar ji duto kata odundu (pep) mochung mar pi,*

W4. tubewell or borehole/*Bur mar pi matut mokuny gi masin (kisima)*

w5. - protected well /*Bur mar pi ma Okuny ma tut ma oriti’*

w6. – unprotected well / *Bur mar pi ma okuny ma tut ma ok oriti’*

W7. – protected spring/ *Soko mar pi moriti*

W7A. – unprotected spring/*Soko mar pi ma ok oriti* *(i.e. springs without protection like box or concrete walls shown in protected spring pictures) Kuom ranyisi, ma onge kod gik ma oritgi godo kaka boxi kata kor ma omuon kod kokoto gi simiti ma oket kaka ranyisi e pichni go.*

W8. rainwater /*Pi Koth*

w9. bottled water/*Pi Chupa*

w11. tanker truck /*Tange mar Lori*

w10. cart with small tank/ *Tange matin mar kanyna*

w12a– free-flowing river or stream/*Pi Aora Mamol*

W12B-stagnant water in dam, pond or lake/*Pi mochung’ mar yawo, dago kata nam*

W12C– canal or irrigation channel/ *Pi mar riwa/oula kata riwa mag miyo ni cham pi (rigesen)*

other: specifY _____________ *Machielo –wache adimba*

**Q2bR1**. **Source of piped water during the wet season.**

Utility

Borehole

Lakewater system

Don’t know

**Q2c. What are the water sources your household uses for cooking during the dry season?**

W1. Piped water – into dwelling/*Pi odundu (pep) Ma odonjo nyaka kar jot*

W2. piped to yard or plot/*Pi odundu (pep) mantie e plot*

W3. public tap or standpipe/*Freji mar ji duto kata odundu (pep) mochung mar pi,*

W4. tubewell or borehole/*Bur mar pi matut mokuny gi masin (kisima)*

w5. - protected well /*Bur mar pi ma Okuny ma tut ma oriti’*

w6. – unprotected well / *Bur mar pi ma okuny ma tut ma ok oriti’*

W7. – protected spring/ *Soko mar pi moriti*

W7A. – unprotected spring/*Soko mar pi ma ok oriti* *(i.e. springs without protection like box or concrete walls shown in protected spring pictures) Kuom ranyisi, ma onge kod gik ma oritgi godo kaka boxi kata kor ma omuon kod kokoto gi simiti ma oket kaka ranyisi e pichni go.*

W8. rainwater /*Pi Koth*

w9. bottled water/*Pi Chupa*

w11. tanker truck /*Tange mar Lori*

w10. cart with small tank/ *Tange matin mar kanyna*

w12a– free-flowing river or stream/*Pi Aora Mamol*

W12B-stagnant water in dam, pond or lake/*Pi mochung’ mar yawo, dago kata nam*

W12C– canal or irrigation channel/ *Pi mar riwa/oula kata riwa mag miyo ni cham pi (rigesen)*

other: specifY _____________ *Machielo –wache adimba*

**Q2d. What are the water sources your household uses for cooking during the wet season?**

W1. Piped water – into dwelling/*Pi odundu (pep) Ma odonjo nyaka kar jot*

W2. piped to yard or plot/*Pi odundu (pep) mantie e plot*

W3. public tap or standpipe/*Freji mar ji duto kata odundu (pep) mochung mar pi,*

W4. tubewell or borehole/*Bur mar pi matut mokuny gi masin (kisima)*

w5. - protected well /*Bur mar pi ma Okuny ma tut ma oriti’*

w6. – unprotected well / *Bur mar pi ma okuny ma tut ma ok oriti’*

W7. – protected spring/ *Soko mar pi moriti*

W7A. – unprotected spring/*Soko mar pi ma ok oriti* *(i.e. springs without protection like box or concrete walls shown in protected spring pictures) Kuom ranyisi, ma onge kod gik ma oritgi godo kaka boxi kata kor ma omuon kod kokoto gi simiti ma oket kaka ranyisi e pichni go.*

W8. rainwater /*Pi Koth*

w9. bottled water/*Pi Chupa*

w11. tanker truck /*Tange mar Lori*

w10. cart with small tank/ *Tange matin mar kanyna*

w12a– free-flowing river or stream/*Pi Aora Mamol*

W12B-stagnant water in dam, pond or lake/*Pi mochung’ mar yawo, dago kata nam*

W12C– canal or irrigation channel/ *Pi mar riwa/oula kata riwa mag miyo ni cham pi (rigesen)*

other: specifY _____________ *Machielo –wache adimba*

**Q2e.Do you have any livestock?**

Yes/No

if no, skip Q2h

**Q2f. What are the water sources your household uses for watering livestock during the dry season?**

W1. Piped water – into dwelling/*Pi odundu (pep) Ma odonjo nyaka kar jot*

W2. piped to yard or plot/*Pi odundu (pep) mantie e plot*

W3. public tap or standpipe/*Freji mar ji duto kata odundu (pep) mochung mar pi,*

W4. tubewell or borehole/*Bur mar pi matut mokuny gi masin (kisima)*

w5. - protected well /*Bur mar pi ma Okuny ma tut ma oriti’*

w6. – unprotected well / *Bur mar pi ma okuny ma tut ma ok oriti’*

W7. – protected spring/ *Soko mar pi moriti*

W7A. – unprotected spring/*Soko mar pi ma ok oriti* *(i.e. springs without protection like box or concrete walls shown in protected spring pictures) Kuom ranyisi, ma onge kod gik ma oritgi godo kaka boxi kata kor ma omuon kod kokoto gi simiti ma oket kaka ranyisi e pichni go.*

W8. rainwater /*Pi Koth*

w9. bottled water/*Pi Chupa*

w11. tanker truck /*Tange mar Lori*

w10. cart with small tank/ *Tange matin mar kanyna*

w12a– free-flowing river or stream/*Pi Aora Mamol*

W12B-stagnant water in dam, pond or lake/*Pi mochung’ mar yawo, dago kata nam*

W12C– canal or irrigation channel/ *Pi mar riwa/oula kata riwa mag miyo ni cham pi (rigesen)*

other: specifY _____________ *Machielo –wache adimba*

**Q2g. What are the water sources your household uses for watering livestock during the wet season?**

W1. Piped water – into dwelling/*Pi odundu (pep) Ma odonjo nyaka kar jot*

W2. piped to yard or plot/*Pi odundu (pep) mantie e plot*

W3. public tap or standpipe/*Freji mar ji duto kata odundu (pep) mochung mar pi,*

W4. tubewell or borehole/*Bur mar pi matut mokuny gi masin (kisima)*

w5. - protected well /*Bur mar pi ma Okuny ma tut ma oriti’*

w6. – unprotected well / *Bur mar pi ma okuny ma tut ma ok oriti’*

W7. – protected spring/ *Soko mar pi moriti*

W7A. – unprotected spring/*Soko mar pi ma ok oriti* *(i.e. springs without protection like box or concrete walls shown in protected spring pictures) Kuom ranyisi, ma onge kod gik ma oritgi godo kaka boxi kata kor ma omuon kod kokoto gi simiti ma oket kaka ranyisi e pichni go.*

W8. rainwater /*Pi Koth*

w9. bottled water/*Pi Chupa*

w11. tanker truck /*Tange mar Lori*

w10. cart with small tank/ *Tange matin mar kanyna*

w12a– free-flowing river or stream/*Pi Aora Mamol*

W12B-stagnant water in dam, pond or lake/*Pi mochung’ mar yawo, dago kata nam*

W12C– canal or irrigation channel/ *Pi mar riwa/oula kata riwa mag miyo ni cham pi (rigesen)*

other: specifY _____________ *Machielo –wache adimba*

**Q2h. W****hat are the water sources your household uses for other domestic uses (e.g washing clothes) during the dry season?**

**(***See picture guide for help on identifying each type of source (W1, W2…..W12 labels are used on pictures).*

*Q2d. Pi ma joodi tiyogo ahinya’ kuom modho uomo kanye? (Rang pichni moketi mondo okonyi yango kit kuonde ma iomoe pige- pichnigo oyang kod ranyisi W1, W2…..W12)*

W1. Piped water – into dwelling/*Pi odundu (pep) Ma odonjo nyaka kar jot*

W2. piped to yard or plot/*Pi odundu (pep) mantie e plot*

W3. public tap or standpipe/*Freji mar ji duto kata odundu (pep) mochung mar pi,*

W4. tubewell or borehole/*Bur mar pi matut mokuny gi masin (kisima)*

w5. - protected well /*Bur mar pi ma Okuny ma tut ma oriti’*

w6. – unprotected well / *Bur mar pi ma okuny ma tut ma ok oriti’*

W7. – protected spring/ *Soko mar pi moriti* W7A. – unprotected spring/*Soko mar pi ma ok oriti* *(i.e. springs without protection like box or concrete walls shown in protected spring pictures) Kuom ranyisi, ma onge kod gik ma oritgi godo kaka boxi kata kor ma omuon kod kokoto gi simiti ma oket kaka ranyisi e pichni go.*

W8. rainwater /*Pi Koth*

w9. bottled water/*Pi Chupa*

w11. tanker truck /*Tange mar Lori*

w10. cart with small tank/ *Tange matin mar kanyna*

w12a– free-flowing river or stream/*Pi Aora Mamol*

W12B-stagnant water in dam, pond or lake/*Pi mochung’ mar yawo, dago kata nam*

W12C– canal or irrigation channel/ *Pi mar riwa/oula kata riwa mag miyo ni cham pi (rigesen)*

other: specifY _____________ *Machielo –wache adimba*

**Q2i. What are the water sources your household uses for other domestic uses (e.g washing clothes) during the wet season?**

**(***See picture guide for help on identifying each type of source (W1, W2…..W12 labels are used on pictures).*

*Q2d. Pi ma joodi tiyogo ahinya’ kuom modho uomo kanye? (Rang pichni moketi mondo okonyi yango kit kuonde ma iomoe pige- pichnigo oyang kod ranyisi W1, W2…..W12)*

W1. Piped water – into dwelling/*Pi odundu (pep) Ma odonjo nyaka kar jot*

W2. piped to yard or plot/*Pi odundu (pep) mantie e plot*

W3. public tap or standpipe/*Freji mar ji duto kata odundu (pep) mochung mar pi,*

W4. tubewell or borehole/*Bur mar pi matut mokuny gi masin (kisima)*

w5. - protected well /*Bur mar pi ma Okuny ma tut ma oriti’*

w6. – unprotected well / *Bur mar pi ma okuny ma tut ma ok oriti’*

W7. – protected spring/ *Soko mar pi moriti* W7A. – unprotected spring/*Soko mar pi ma ok oriti* *(i.e. springs without protection like box or concrete walls shown in protected spring pictures) Kuom ranyisi, ma onge kod gik ma oritgi godo kaka boxi kata kor ma omuon kod kokoto gi simiti ma oket kaka ranyisi e pichni go.*

W8. rainwater /*Pi Koth*

w9. bottled water/*Pi Chupa*

w11. tanker truck /*Tange mar Lori*

w10. cart with small tank/ *Tange matin mar kanyna*

w12a– free-flowing river or stream/*Pi Aora Mamol*

W12B-stagnant water in dam, pond or lake/*Pi mochung’ mar yawo, dago kata nam*

W12C– canal or irrigation channel/ *Pi mar riwa/oula kata riwa mag miyo ni cham pi (rigesen)*

other: specifY _____________ *Machielo –wache adimba*

**Q2j. During the dry season do you draw drinking water from the same source that livestock use?**

YES

NO

Don’t know

**Q2k. During the wet season do you draw drinking water from the same source that livestock use?**

YES

NO

Don’t know

### WATER TREATMENT AND STORAGE

**Q3a. Who is responsible for making decisions on water handling, management or safety once the water is in the household?** *Note: respondents should be aged 18 years or over. Do not ask those younger than this age to respond to the survey.*

Respondent

Someone else

If respondent, skip to Q4

**Q3b.What is the relationship of the person responsible to the household head?**

(Head/Spouse/Son/Daughter/Brother/Sister/Uncle/Aunt/Nephew/Niece/Grandchild/Other)

**Q4. Do you have a water reservoir outside the house within the compound?**

Yes

No

**If no, skip to Q6.**

**Q4a. Do you have a borehole or well within your compound?**

Borehole

Hand dug well

Neither

**Q4b.  Do you have a tank, drum or other container outside the house but within the compound for storing rainwater or piped water?**

Drum – for rainwater

Overhead tank – for rainwater

Underground tank – for rainwater

Tank at ground level – for rainwater

Other ***(specify)*** – for rainwater

Drum – for piped water

Overhead tank – for piped water

Underground tank – for piped water

Tank at ground level – for piped water

Other ***(specify)*** – for piped water

None

**Q4c.Number of drum/overhead tank/ground level/underground tank_________________?**

**Q4d.Capacity in litres of drum/overhead tank/ground level/underground tank____________?**

**Q5. Do you ever run out of drinking water from this reservoir during the dry season?**

Yes

No

Don’t know

**Q6. Do you store the water that you use for drinking?**

YES

NO

If yes, skip to Q8

**Q7. What water do you use for drinking?**

Drums

Piped water

Tank

Other-------specify

**Q8. Do you have any water stored now?**

Yes

No

If yes, skip to Q10

**Q9a.How long ago (in hours) did the water run out**? --------------------(type in)

**Q9b. If your youngest child needed drinking water now, what would you do to get the water?**

Go to my neighbour/relative

Buy from shop/kiosk

Go fetch it

Nothing

Other_________(specify)

**Q10. Does your youngest child drink from the same storage?**

Yes,

NO, skip to Q12

**Q11. Did you do anything to the water to make it safer for drinking?**

Yes

No, skip to Q14

Don’t know, skip to Q14

**Q12. Did you do anything to the water to make it safer to drink for children under under 5 years?**

Yes

No, skip to Q14

Don’t know, skip to Q14

**Q13.What did you do to make the water safer for drinking?** *(Record all mentioned)*

BOIL/ *Chwako*

ADD BLEACH / CHLORINE/ Water guard *Keto klorin*

ADD A WATER COAGULANT (e.g. alum)/ *Keto yadh pi (kaka Alam)*

STRAIN THROUGH A CLOTH/ SIEVE *Chungo pi gi Law/nanga*

USE WATER FILTER (CERAMIC / SAND / COMPOSITE ETC)/ *Tiyo gi rachungi mar pi (kidi motuchotuch mar chungo pi/Kuoyo/riwo Kuoyo Kod kidi motuchotuch gi mamoko).*

SOLAR DISINFECTION/*Keto pi echieng’ mondo oneg kute*.

LET IT STAND TO SETTLE/ *Keto pi mondo ochingre*

OTHER (SPECIFY: ___________________________)/ *Machielo? Wache adimba*.

DON’T KNOW/*Ok ang’eyo*

**Q14. a).Where did the water stored in this container come from?**

W1. Piped water – into dwelling/*Pi odundu (pep) Ma odonjo nyaka kar jot*

W2. piped to yard or plot/*Pi odundu (pep) mantie e plot*

W3. public tap or standpipe/*Freji mar ji duto kata odundu (pep) mochung mar pi,*

W4. tubewell or borehole/*Bur mar pi matut mokuny gi masin (kisima)*

w5. - protected well /*Bur mar pi ma Okuny ma tut ma oriti’*

w6. – unprotected well / *Bur mar pi ma okuny ma tut ma ok oriti’*

W7. – protected spring/ *Soko mar pi moriti* W7A. – unprotected spring/*Soko mar pi ma ok oriti* *(i.e. springs without protection like box or concrete walls shown in protected spring pictures) Kuom ranyisi, ma onge kod gik ma oritgi godo kaka boxi kata kor ma omuon kod kokoto gi simiti ma oket kaka ranyisi e pichni go.*

W8. rainwater /*Pi Koth*

w9. bottled water/*Pi Chupa*

w11. tanker truck /*Tange mar Lori*

w10. cart with small tank/ *Tange matin mar kanyna*

w12a– free-flowing river or stream/*Pi Aora Mamol*

W12B-stagnant water in dam, pond or lake/*Pi mochung’ mar yawo, dago kata nam*

W12C– canal or irrigation channel/ *Pi mar riwa/oula kata riwa mag miyo ni cham pi (rigesen)*

other: specifY _____________ *Machielo –wache adimba*

**Q14b) Did you experience any disruptions to your piped water supply since this time yesterday?**

YES

NO----skip to Q15

DON’T KNOW…...skip to Q15

**Q14c). How long ago did the water come back on again?**

Last half hour

Last hour

Last four hours

More than four hours ago

Don’t Know

Not yet

**Q14d. In the past four weeks, because of a lack of water, have you had to reduce how much water you use?**

Yes

No

Don’t know

**If yes, for which uses?** *(Checkbox)*

Drinking

Cooking or preparing food

Bathing and washing of body / hands

Watering crops or vegetables

Watering animals

**Q14e. In the past four weeks, because of problems accessing water, have you had to change the time when you do any of the following?**

Yes

No

Don’t know

**If yes,** *(checkbox)*

Drinking

Cooking or preparing food

Bathing and washing of body / hands

Watering crops or vegetables

Watering animals

**Q15.Is the source of the stored water within the compound?**

Yes

NO, skip to Q16b

**Q16a.Do you ever get water from outside the compound?**

Yes

No-----skip to Q22

**Q16b. In which village is the water source located?**

Ongielo

Kaminogedo

Ndwara/Nyangoma

Lwak

Sinogo

Sangla

Wangarot A

Siger

Rambugu B

Got Bondo

Other------------skip to Q17d

**Q17c.Local name of water in the selected village_________________? (select from drop list)**

**Q17d.Name of village-----------------------?**

**Q17e.Local name of the water source----------------------?**

**WATER FETCHING**

**18a. On average, have you fetched water from this source during the last month? (***Record one option below)*

Daily

Weekly

Monthly

Don’t know---------- Skip to Q20

No--------- skip to Q20

**Q18b.Number of trips per day/week/month…………?**

**Q18c. How long does it take per trip……….? (***Number of hours or minutes***)**

**Q18d. Number of containers per trip………….?**

**Q18e. Size of each container in litres**…………….? (*e.g 20 for 20 litre container*)

**Q19. Do you pay for the water you fetch?**

Yes

No

Don’t know

Q19b. **If yes, what is the cost per container?** ------type in (*if no amount paid, enter 0)*

**Q20. Who is responsible for fetching water inyour household?**

**(Spouse/Son/Daughter/Brother/Sister/Uncle/Aunt/Nephew/Niece/Grandchild/Other)** *Note: respondents should be aged 18 years or over. Do not ask those younger than this age to respond to the survey.*

**Q21a.What means of transport do you use to fetch water?**

On foot

Motorbike

Bicycle

Hand-cart

Donkey

Other-------------------specify

**Q21b. How much does it cost you to get the water per trip by the means selected_____________? (***Enter 0 if no cost incurred)*

**STRUCTURED OBSERVATIONS AND LIVESTOCK IN THE COMPOUND AND POTENTIAL FOR LIVESTOCK CONTACT WITH STORED WATER**

**Q22 Are you conducting the interview inside the respondent’s home?**

YES

NO, skip to Q25a

**Q23. Did you observe any of the following animals inside the home during the interview?** *(Tick all that apply)*

Poultry

Dogs

Goats

Cows

Sheep

Donkeys

None

OTHER. Specify: ____________

**Q24. Is there any evidence of animals accessing the house or the water source***? (Observe but do not ask. Look for e.g Animal droppings, animal tracks, feathers, etc)*

YES

NO

**Q25a. Do you own any poultry?**

YES

NO, skip to Q27a

**Q25b. If yes, are they confined (e.g. in a coop):**

All the time

Sometimes

Never

**Q26. Do chicken spend the night in the same place where drinking water is kept?**

YES

NO

### CONFOUNDERS: SANITATION AND HYGIENE BEHAVIOUR

*Gik nyalo miyo dwoko mar nonro ok bed kaka jonro paro: Ler mar aluora kod Kaka tim tudore kod rito ler.*

**Q27a. What kind of toilet facility do members of your household usually use?**

s1A. flush / pour flush toilet / *igoyo/olo pi ka dhi e*

s4. Composting toilet/*S4 Choo mo iiko*

S5. Ventilated improved pit latrine (VIP): *S5. Choo man kod kuma golo muya oko ma olos maber ma oingo moko*

s6. Pit latrine with slab/*S6 Choo mar bur man kod dier ot ma olos kod simiti, kokoto gi kuoyo (slab),*

s7. Pit latrine without slab/open pit/*Choo mokuny maonge slab/Bur ma ndhoge ni nono*

s8. Bucket/*Ndoo*

s9. Hanging toilet / hanging latrine/*Choo ma iliero to iwito losruok oko*

s10. No facilities or bush or field/*Onge kama watere/bungu/pap*

Other: specify ___________________________ *Machielo: wach adimba*

*See picture guide for help on identifying each type of source (S1, S2..S10 labels are used on pictures.*

*(Kaponi otiyo gi choo migoyo kata miolo pi to tem penjo matut: Igoye kochomo kanye? Rang ane pichni mondo okonyi yango kit kuma giaye (S1, S2..S10 lebo otigo e pichego).*

*If FLUSH/POUR FLUSH go to Q27b; If BUCKET go to Q27c; otherwise skip to Q28.*

**Q27b. Where does it flush to?**

*Q27b. Joodi tiyo ga gi kit choo machalo nade?*

S1. Piped sewer system/*S1* *odundu (pep) mar keyo minyaga*

S2. Septic tank/*S2 tangi mar kano minyaga*

S3. Pit latrine/*S3* *choo mar bur*

S3a. Elsewhere/*S3* *a kuonde mamoko*

S3B. Unknown place /not sure / do not know where/*S3B kama maok onge’/ok en gadiera/ ok ongeyo ni gidhi kanye.*

**Q27c. Where do you empty the bucket?**

Pit latrine *choo mar bur*

RUBBISH PIT

WATER COURSE

FARMLAND

Elsewhere/*S3* *a kuonde mamoko*

Unknown place /not sure / do not know where/*S3B kama maok onge’/ok en gadiera/ ok ongeyo ni gidhi kanye.*

**Q28. The last time your youngest child passed stool, what was done to dispose of the stool?**

*Q28.. Chieng mogik manyo nyathini matin mogik olosore, ne itimo ‘nango’ mondo losruokne ogol oko mondo owiti?*

Child used toilet/latrine/ *Nyathi ne otiyo kod choo*

Put/rinsed into toilet or latrine/*Ne oketo/alao ei choo*

Put/rinsed into drain or ditch/*Ne keto/alaw e oula kata e bur*

Thrown into garbage/ *Ne owito ei yugi (taka)*

Buried/*Ne Oiko piny*

Left in the open/ *Ne oweyo aweya oko*

Other (specify)/*Machielo (wach adimba)*

DO NOT KNOW/*Ok ong’eyo*

**Q29. How do members of your household wash their hands?**

*Q.29. Joodi luoko lwetgi nade?*

WITH SOAP OR DETERGENT (BAR, LIQUID, POWDER, PASTE)/ *Gi sabun kata kit gir golo chilo sabun (sabun mar miti, sabun mar pi, sabun mar poda, katasabun modual)*

WITH ASH, MUD, OR SAND/*Gi buru, chuodho kata kuoyo*

WITH WATER/*gi pi*

OTHER – SPECIFY: _____________*Machielo _________________ Wach adimba*

DON’T KNOW/ *Ok ong’eyo*

*If WITH WATER, ASH/MUD/SAND, OR DON’T KNOW skip to Q31*

*Ka gi pi kata ok ongéyo, to chikri mondo ichopi e penjo namba piero adek kod a biriyo (39*)

**Q30. Can you show me the soap or detergent you use to wash your hands?**

*Q30. Be inyalo siemonae sabun kata kit sabun mitiyogo kuom luoko lwedo?*

SOAP OR DETERGENT (BAR, LIQUID, POWDER, PASTE) ASH, MUD, OR SAND (*sabun miti, mar pi, mar poda, kata modual) buru, chuodho, kata kuoyo.*

NONE OR IT IS FINISHED

PERMISSION NOT GRANTED TO SEE/ *Thuolo ok ochiw mar neno*.

**Q31. When was the last time you washed your hands?** *(Do not read out the different answers below- just tickthe one or two situations that the respondent mentions).*

*Q31. En kinde mane ma joodi gi luoko e lwetgi?*

AFTER TOILET/*Bang’ dhi e choo*

BEFORE COOKING/*Kapok otedi*

BEFORE EATING/*Kapok ochiem*

AFTER CLEANING BABY’S BACKSIDE/ *Bang’ yweyo sianda nyathi*

BEFORE FEEDING BABY/*Kapok opidh nyathi chiemo*

AFTER EATING

OTHER (SPECIFY): ___________________*Machielo (Wach adimba)*

**Q32. The time before that, when did you wash your hands?** *(Do not read out the different answers below- just tickthe one or two situations that the respondent mentions).*

*Q32. En kinde mane ma joodi gi luoko e lwetgi?*

AFTER TOILET/*Bang’ dhi e choo*

BEFORE COOKING/*Kapok otedi*

BEFORE EATING/*Kapok ochiem*

AFTER CLEANING BABY’S BACKSIDE/ *Bang’ yweyo sianda nyathi*

BEFORE FEEDING BABY/*Kapok opidh nyathi chiemo*

AFTER EATING

OTHER (SPECIFY): ___________________*Machielo (Wach adimba)*

**HOUSEHOLD STORAGE: WATER TREATMENT AND STORAGE**

**Q33. Please show me where you store the water that you use for drinking in the home?**

*(Ask about water storage if you are not allowed to observe the stored water directly)*

*(Penj kuma ikano e pi ka thuolo onge ma inyalo ng’iyogo matut maling’ ling’kama okane pi)*

OBSERVED

NOT OBSERVED/NO PERMISSION TO SEE ------------skip to Q35

NOT OBSERVED, OTHER REASON -----------------------skip to Q35

**Q34. Select the type(s) of storage in the list below (if more than one method used, select all of those that are used).**

*Q34. Luor (gi kalam) achiel kuom yore mikanogo pi mopang piny gi (Kaponi yore mikanogo pi ng’eny moingo achiel, to luor achiel ma itiyogo kuom kano pi modho mar nyathi)*

SMALL CONTAINER (e.g. 20 litre jerrycan/pot or smaller)/*Gima matin ma iting’o godo pi (kaka Kube/Mbugru mar lita 20 kata matin)*

LARGE CONTAINER (larger than 20 litre jerrycan/pot, up to 100 litres in size)/*Gima duong’ ma iting’o godo Pi (Kaka Kube/Mbugru moingo lita piero ariyo (20), kata nyaka lita mia achiel (100))*

TANK (larger than 100 litres)/ *Tangi (Moingo lita mia achiel (100))*

WATER STORED IN CONTAINERS BOUGHT AT SHOP (E.G. COMMERCIAL BOTTLED WATER)

*Pi okan e gima iting’o go pi mongi’ew e duka (kaka Pi ohala mar chupa mong’iew)*

OTHER (SPECIFY: __________________) *Machielo (Wach adimba)*.

DON’T KNOW / *ok ang’eyog*

**Q35. When was this container (used to store drinking water in the home) last cleaned?**

*Q35. Gir ting’o pi ni niluoko mogik karango’?*

TODAY/*Kawuono*

YESTERDAY/*Nyoro*

IN THE LAST WEEK/*Juma mokalo*

IN THE LAST MONTH/*Dwe mokalo*

LONGER THAN THIS/*Ose gwaro matin*

NEVER/*Ok luokega /*

DON’T KNOW/ *ok ang’eyo*

*If answer is ‘NEVER’ or ‘DON'T KNOW, skip to Q36*

***Q36.*** **What was used to clean container? (***Tick all that apply)*

Water only

Water and Detergent

Water and abrasive (cloth, net or sand)

Water, detergent and abrasive (cloth, net or sand)

Don’t know

**Q37. Which part of the container was cleaned?** *(tick all that apply)*

Inside

Outside

Lid

Don’t know

**Q38. Are you collecting a sample now?**

(*Request to come back later to collect a sample)*

Yes

No

### HOUSEHOLD STORAGE: OBSERVATIONS OF HOUSEHOLD WATER STORAGE CONTAINER AND BEHAVIOUR FETCHING STORED WATER

***Q39****.* **Is the vessel accessible for animals or birds?**

YES

NO

*Q40***. Is the vessel kept above waist height?**

YES

NO

***Q41. What is the vessel made of? (select one only)***

PLASTIC/*Plastik*

METAL/*Chuma*

CLAY/*Loo*

WOOD/*Bao*

CERAMIC/GLASS

OTHER – SPECIFY: ____________ / *Machielo - Wach adimba*

*Q42*. **Do they have a lid currently on the storage vessel or is it covered?** *(tick ‘NO’ if the lid is there but not in place covering the container)*

YES

NO

***Q43. Are there signs of dirt on the container or lid?***

YES

NO

**Q44. Is the water container kept indoor or outdoor*?***

Indoor

Outdoor

*If OUTDOOR, skip to Question 45.*

**Q45. If indoors, where?**

Kitchen

Bedroom

Living room

Other (specify)

Specify the site (part of the room): _____________

**Q46. Specify the site (part of the room where the container is kept)**

Next to the door

Away from the door

*Q47***. Can you bring us a sample of the water stored in this container?**

YES – PERMISSION GRANTED

NO – PERMISSION NOT GRANTED---------(Questionnaire ends)

NO STORED WATER AVAILABLE ---------(Questionnaire ends)

**Q48. Can you observe how the respondent draws water from the container?**

YES

NO*--------***skip to Q51**

*Observe the following whilst water is being fetched.*

*Ngiy ane matut maling’ ling’ gik ma ipenjo bang ma esama ituomo pi e kama okanee.*

**Q49***.* **Does the respondent wash his/her hands before fetching water?**

YES

NO

NOT ABLE TO OBSERVE

**Q50. Is there any contact between water and hands as the respondent draws water from the vessel?**

*Q48. Be nitie tudruok moro amora e kind lwedo kod pi ka jaduok penjo tuomo pi ei gir kano pi no?*

YES – CONTACT WITH HANDS/*Eee- Nitie tudruok mar Lwedo gi pi*

NO – NO CONTACT WITH HANDS/ *Ooyo- Onge tudruok moro mar pi gi lwedo*.

NOT ABLE TO OBSERVE/ *Ne ok anyal ng’iyo matut maling’ ling’*

**Q51. How does the respondent take water from the vessel?**

*Q49. Be jaduok penjo tiyo gi okombe kata gima nigi wende (bade) mabor ma inyalo dumbe go ei gi ma okan e pi?*

CUP WITH HANDLE

CUP WITHOUT HANDLE

JUG

INCLINED JERRYCAN

DIPPER (WITH LONG HANDLE)

TAP

OTHER – SPECIFY: __________________

NOT ABLE TO OBSERVE/ *Ne ok anyal ngiyo maling’ ling’*

*If TAP, OTHER, JERRYCAN or NOT ABLE TO OBSERVE, skip to Q50.*

*Kaponi Ooyo kata ok inyal ngiyo matut maling’ ling’, to kal nyaka namba Q50.*

**Q52. Where do they put the cup, dipper, or jug after using it***?*

*Q50. Ka gisetiyo gi okombe kata gima idumbo ka ituomo godo pi no to gikete kanye?*

CUP/DIPPER/JUG IS PUT BACK IN THE WATER IN THE VESSEL

CUP/DIPPER/JUG IS PLACED ON OR TIED TO OUTSIDE OF THE VESSEL*.*

CUP/DIPPER/JUG IS PUT SOMEWHERE ELSE

### TAKING THE HOUSEHOLD WATER SAMPLE

*Kawo pi matin mar ranyisi nonro*

**Q53a. Pour water into sample bottle and record sample bottle ID: 🞏🞏🞏**

*(Enter atleast 9 characters):*

**Q53b. Confirm sample ID---------------------------**

*(Enter atleast 9 characters)*

**Q54a. Look at the sample of water and tick all observations that you see**

Coloured water

Cloudy water

Floating/Suspended particles

Clear water

**Q54 b). Level of chlorine **(in ppm or mg/l)****

### **CHILD STORAGE: OBSERVATIONS OF HOUSEHOLD WATER STORAGE CONTAINER FOR THE YOUNGEST CHILD AND BEHAVIOUR FETCHING STORED WATER**

**(*In a case where the child drinks from a different storage, answer the the questions that follow)***

**Q55. Please show me where you store the water that you would normally give to the youngest child to drink?**

*(Ask about water storage if you are not allowed to observe the stored water directly)*

*(Penj kuma ikano e pi ka thuolo onge ma inyalo ng’iyogo matut maling’ ling’kama okane pi)*

OBSERVED

NOT OBSERVED/NO PERMISSION TO SEE ------------skip to Q55

NOT OBSERVED, OTHER REASON -----------------------skip to Q55

**Q56. Type of storage done (**i*f more than one method used, select the one used most recently to give to a child to drink)***.**

SMALL CONTAINER (e.g. 20 litre jerrycan/pot or smaller)/Gima matin ma iting’o godo pi (kaka Kube/Mbugru mar lita 20 kata matin)

LARGE CONTAINER (larger than 20 litre jerrycan/pot, up to 100 litres in size)/Gima duong’ ma iting’o godo Pi (Kaka Kube/Mbugru moingo lita piero ariyo (20), kata nyaka lita mia achiel (100))

TANK (larger than 100 litres)/ Tangi (Moingo lita mia achiel (100))

WATER STORED IN CONTAINERS BOUGHT AT SHOP (E.G. COMMERCIAL BOTTLED WATER)

Pi okan e gima iting’o go pi mongi’ew e duka (kaka Pi ohala mar chupa mong’iew)

OTHER (SPECIFY: __________________) Machielo (Wach adimba).

DON’T KNOW / ok ang’eyog

**Q57. When was this** **container used to store water that you would give to the youngest child last cleaned?**

TODAY/Kawuono

YESTERDAY/Nyoro

IN THE LAST WEEK/Juma mokalo

IN THE LAST MONTH/Dwe mokalo

LONGER THAN THIS/Ose gwaro matin

NEVER/Ok luokega /

DON’T KNOW/ ok ang’eyo

If answer is ‘NEVER’ or ‘DON'T KNOW, skip to Q58

**Q58. What was used to clean the container used to store water that you would give to the youngest child? (Tick all that apply)**

Water only

Water and Detergent

Water and abrasive (cloth, net or sand)

Water, detergent and abrasive (cloth, net or sand)

Don’t know

**Q59. Which part of the container used to store water that you would give to the youngest child was cleaned? (tick all that apply)**

Inside

Outside

Lid

Don’t know

**Q60. Are you collecting a sample for the separate storage for the youngest child now?**

*(Request to come back later to collect a sample)*

Yes

No

*If No, questionnaire ends*

**Q61. Is the vessel used to store water that you would give to the youngest child accessible for animals or birds?**

YES

NO

**Q62. Is the vessel used to store water that you would give to the youngest child kept above waist height?**

YES

NO

**Q63. What is the vessel used to store water that you would give to the youngest child made of? (select one only)**

PLASTIC/Plastik

METAL/Chuma

CLAY/Loo

WOOD/Bao

CERAMIC/GLASS

OTHER – SPECIFY: ____________ / Machielo - Wach adimba

**Q64. Do they have a lid currently on the storage vessel used to store water that you would give to the youngest child or is it covered? (tick ‘NO’ if the lid is there but not in place covering the container)**

YES

NO

**Q65. Are there signs of dirt on the container used to store water that you would give to the youngest child or lid?**

YES

NO

**Q66. Is the water container used to store water that you would give to the youngest child kept indoor or outdoor?**

INDOOR

OUTDOOR

If OUTDOOR, skip to Question 67.

**Q67. If indoors, where is the container used to store water that you would give to the youngest child kept?**

Kitchen

Bedroom

Living room

Other (specify)

Specify the site (part of the room): _____________

**Q68. Specify the site (part of the room where the container is kept)**

Next to the door

Away from the door

**Q69. Can you bring us a sample of the water stored in this container as though for the youngest child to drink?**

YES – PERMISSION GRANTED

NO – PERMISSION NOT GRANTED---------(Questionnaire ends)

NO STORED WATER AVAILABLE ---------(Questionnaire ends)

**Q70. Can you observe as/how the respondent draws water from the container used to store water given to the youngest child?**

YES

NO--------skip to Q73

***Observe the following whilst water given to the youngest child is being fetched.***

**Q71. Does the respondent wash his/her hands before fetching water that is given to the youngest child?**

YES

NO

NOT ABLE TO OBSERVE

**Q72. Is there any contact between water and hands as the respondent draws water from the vessel used to store water given to the youngest child?**

YES – CONTACT WITH HANDS/Eee- Nitie tudruok mar Lwedo gi pi

NO – NO CONTACT WITH HANDS/ Ooyo- Onge tudruok moro mar pi gi lwedo.

NOT ABLE TO OBSERVE/ Ne ok anyal ng’iyo matut maling’ ling’

**Q73. How does the respondent take water from the vessel used to store water that you would give to the youngest child?**

CUP WITH HANDLE

CUP WITHOUT HANDLE

JUG

INCLINED JERRYCAN

DIPPER (WITH LONG HANDLE)

TAP

OTHER – SPECIFY: __________________

NOT ABLE TO OBSERVE/ Ne ok anyal ngiyo maling’ ling’

If TAP, OTHER, JERRYCAN or NOT ABLE TO OBSERVE, skip to Q73.

**Q74. Where do they put the cup, dipper, or jug after using it to draw water given to the youngest child?**

CUP/DIPPER/JUG IS PUT BACK IN THE WATER IN THE VESSEL

CUP/DIPPER/JUG IS PLACED ON OR TIED TO OUTSIDE OF THE VESSEL.

CUP/DIPPER/JUG IS PUT SOMEWHERE ELSE

TAKING THE HOUSEHOLD WATER SAMPLE

Kawo pi matin mar ranyisi nonro

**Q75a. Pour water into sample bottle and record sample bottle ID: (for the stored water given to the youngest child to drink)**

*(Enter atleast 9 characters)*

**Q75b. Confirm sample ID (for stored water given to the youngest child to drink) ---------------------------**

*(Enter atleast 9 characters)*

**Q76 a). Look at the sample of water given to the youngest child and tick all observations that you see**

Coloured water

Cloudy water

Floating/Suspended particles

Clear water

**Q76 b). Level of chlorine **(in ppm or mg/l)** of the water given to the youngest child**

**#####END####**
