# Supplementary material for: A mixed methods study to evaluate participatory mapping for rural water safety planning in western Kenya
Source: PLoS One. 2021 Jul 28;16(7):e0255286. doi: 10.1371/journal.pone.0255286 (PMC8318241; doi:10.1371/journal.pone.0255286)
Supplement: S2 Protocol — (DOCX) [file pone.0255286.s008.docx]

**Article Title:** A mixed methods study to evaluate participatory mapping for rural water safety planning in western Kenya

**Journal name**: PLoS ONE

**Names of the authors:**

Joseph Okotto-Okotto, Weiyu Yu, Emmah Kwoba, SM Thumbi, Lorna G. Okotto, Peggy Wanza, Diogo Trajano Gomes da Silva, Jim A. Wright*

*** Corresponding author**: School of Geography and Environmental Science, University of Southampton, UK. Email: [j.a.wright@soton.ac.uk](mailto:j.a.wright@soton.ac.uk)

**S2 Protocol. Participatory Mapping Topic Guide**

**Ite mar 6:** Penjo mar joma nigi lony kuom loso mep ma ji tiyo kanyakla

**Date: _____ / _____ / __________**

**Village / sub-location: ________________**

# Start time: _____: _____

**End time: _____: _____**

**Number of male participants: _________**

**Number of female participants: ____________**

**Names (first names only or usual form of address including nicknames) for participants and seating chart**

**Facilitator’s name: ______________________ Signature and date: ____­­___________________**

**Timekeeper’s/Note taker’s name: _________________ Signature and date: ___________________**

Short questionnaire to be completed by respondents.

**Facilitator’s name: ________________**

**Signature and date: ____­­_____________**

**Timekeeper’s/Note taker’s name: _________________**

**Signature and date: ___________________**

**Q1. What are the main types of source that you use for drinking, cooking or other domestic uses (e.g. laundry, washing) or watering livestock in the dry season?**

*Q1. Kit kuonde ma uomoe pige ma utiyogo ahinya kuom modho, kata tich mar ot kaka luokruok, tedo kod luoko gin mage?*

**Q2. What do you use [WATER SOURCE] for in the dry season?**

*Q2. Pige ma uyudo kuodego utiyogo e yo mane?*

**Q3. What are the main types of source that you use for drinking, cooking or other domestic uses (e.g. laundry, washing) or watering livestock in the wet season?**

*Q3. Kit kuonde ma uomoe pige ma utiyogo ahinya kuom modho, kata tich mar ot kaka luokruok, tedo kod luoko gin mage?*

**Q4. What do you use [WATER SOURCE] for in the wet season?**

*Q4. Pige ma uyudo kuodego utiyogo e yo mane?*

Prompts:

- ***Drinking?*** *Modho?*
- ***Cooking?*** *Tedo?*
- ***Washing?*** *Luoko?*
- ***Bathing?*** *Luokruok?*
- ***Livestock?*** *Jamni?*
- ***Others…..?*** *Mamoko?*

*Have a short discussion about the meaning of contamination*

**Q5. In your view, what are the main potential sources of water contamination in [WATER SOURCE] in the dry season?**

*Q5. Un Kaka upare, gik makelo sum kata chido ne pi e gueng’ u ka aa kanye?*

**Q6. In your view, what are the main potential sources of water contamination in [WATER SOURCE] in the wet season?**

*Q6. Un Kaka upare, gik makelo sum kata chido ne pi e gueng’ u ka aa kanye?*

***Prompts***

- ***Sanitation?*** *(Facilitator to break down sanitation into simpler elements that constitute sanitation) Ler mar aluora kod gik manie ie?(Jatachenro mondo oket gik moko mayot winjo kotenore kod ler mar aluora kod gik manie ie?*
- ***Livestock – where livestock spend more time?*** *(dung, urination, etc) Jamni? (Owuoyo, lach jamni, ekind mamoko)*
- ***Waste / refuse?*** *(Yugi/ochafu/taka?*
- ***Areas where animals are slaughtered****?Kuonde ma iyango’ e jamni?*
- ***Other hazards…..?*** *Midhierni mamoko?*

**Q7. Can you arrange these contamination hazards that we have talked about in order of importance from the most serious to the least serious sources? [Voting/consensus method: thumbprints under different contamination sources]**

*Q7. Kuom midhierni ma kelo sum kata lil mar pi ma wasewuoye go, chan gi uru ane ka oluwore gi pek mag gi kod chadruok magikelo kochakore gi marachie moloyo nyaka maok rach ahinya?*

**Q8. *How long has each hazard been present (of the three most important)?***

*Q8. Kuom midhierni gi be nitie mabet mana kuom kinde matin?*

## Mapping Exercise: using satellite imagery of the community

***Note: This section involves participants recording information about water sources and hazards on transparencies laid over base map satellite images of each village, similar to those seen in Google Maps****.*

*Par: Kae, jogo duto ma wangodo e bura ni biro ndiko weche motenore gi kuma pi wuokie kod midhierni mag pi e kalatasmanyilni moket ewi map kod pichni mogo mag kor yamo mar gweng’ ka gweng’machalre gi manitie e map mowuok e mbui.*

**Q9. What local landmarks are there in this area, such as schools, shops, or churches?**

*Q9. Gin kuonde mage ma mong’ere ahinya e gweng’ u ka, kaka skunde, dukni, kata kanise?*

**Q10. Now let us see if we can find where the geographic landmarks you have just listed are on this photograph, taken from the air.**

*Q10. Koro we wane ane watemu uru kaponi wanyalo yudo gik moko kata kuonde ma onge’re ma uwachogo kaponi wanyalo yudo gi ei epicha mane okaw e kor yamo.*

***[Aerial imagery orientation exercise follows, where participants find local landmarks on a real imagery].***

*Jata bura mondo oler ne jobura kaka ginyalo nge’yo yudo kuonde ma ong’ere e gweng’gi ei picha ma okaw e kor yamo, kaeto omiyogi teko mar yangogi kendo gorogi ei kalatas kalatas manyilni ma oketi e wi picha.*

**Q11. I will now give you pens with different colours. Use them to draw the main roads (brown), key foot paths (red), markets (black rectangle), rivers (light blue) thus using a different colour for each.**

*Q11. Koro adhi miyo u kalembe man kod kido mopogore opogore. Kalambe go udhi goro go yore madongo (rabuor) yore matindo mag tielo (rakwaro), Chirni (boksi marateng), Aoche ( Rambulu ma marmar). Koro kuom moro ka moro utigi kido mopogore opogore.*

**Q12. Now we will look at the aerial photographs again and see if we can identify the locations of water sources in this area and mark or draw them on the Image (photo) using those pens that I have given you.**

*Q12. Koro wadhi ng’iyo pichni mane okaw e kor yamo ka kendo mondo wane ane kaponi wanyalo yango kuonde ma pi wuokie e gweng’ni, kaeto waket kido moro kata wagorgi e pichano kawatiyo gi kalambe mane amiyou ka.*

*[Participants use coloured pens to draw locations of water sources on transparencies fixed over the image (aerial photograph). Different Colours used to denote different types of sources]*

*Jobura tiyo gi kalembe mondo ogor kuonde ma pi wuokie e kalatas manyilni moketi ewi piche mogo e kor yamo. (Kido mopogore opogore mag kalambe mondo otigo kanyiso kuonde mopogore opogore ma pi wuokie)*

**Q13. Can you tell me about the history and management of the wells and boreholes we have just drawn?**

*Q13. Be unyalo ng’isa sigand soknigi kod kaka irito kisimbe ma wagorogi?*

**Prompts:**

- ***Who installed this source?*** *Ng’ama ne guro kar omo pi ni?*
- ***When did they install it?*** *Ne gigure karango’?*
- ***How did they install it?*** *(e.g. by hand? With a hand auger (also known as a vonder rig)?*

*With a borehole drilling rig?) Ere kaka ne gigure? (Gilwedo, Masin mikunyogo gi lwedo? Man kod masin makunyo kisima kaluorore)*

- ***Who manages it now?*** *En nga’ marite?*
- ***If a borehole/well, how deep is it?*** *Kaponi en kisima to tutne romo nadi?*

**Q14. Can you also now show where the contamination hazards that we discussed earlier are currently located on the image (photo)? (Use different shades of red)**

*Q14. Be unyalo ng’iso kuonde ma midhierni mag sum kata makelo ketruok mar pi mane wawuoye moteloka nitie e pichani? (ti uru gi kido mag kalembe ma opogore mobuki ma rakwaro)*

***[Participants use coloured pens to draw and shade locations of water contamination hazards on transparencies fixed over aerial photographs, with at least 8 known locations marked on each transparency to enable subsequent geo-referencing. Colours used to denote different types of hazard]***

*Jobura ma wan godo kae mondo oti gi kalembe mondo ogor kendo obuki kuonde ma sum kata makelo midhierni ne pi e kalatas maler moketi ewi picha mane okaw e kor yamo, koro giket kuonde maok tin ne aboro e kalatas ka kalatas mondo okel yot ne muonogi e map. (Kido mopogore opogore mag kalembe onego otigo mondo ongis kido mopogore opogore mag midhierni).*

**Q15. Are there any other issues about the safety or contamination of water sources that we have not discussed but which you feel are important? If yes, which ones?**

*Q15. Be nitie weche moko koluore gi ong’ala makare mar kuma pi wuokie, kata gima kelo kethruok mar kuma pi wuokie ma ok wagoyoe mbaka/twak to uparoni nigi teko kendo onego wawuoye? Kaponi ee, to gin mage?*
