# Supplementary material for: A mixed methods study to evaluate participatory mapping for rural water safety planning in western Kenya
Source: PLoS One. 2021 Jul 28;16(7):e0255286. doi: 10.1371/journal.pone.0255286 (PMC8318241; doi:10.1371/journal.pone.0255286)
Supplement: Attachment — Submitted filename: Okotto-Okottoetal_Response_ReviewersComments.docx [file pone.0255286.s009.docx]

# Response to Reviewers’ Comments: “A mixed methods study to evaluate participatory mapping for rural water safety planning in western Kenya” (Okotto-Okotto et al; Manuscript Number: PONE-D-20-28371)

Reviewer #1: 

This manuscript introduces a mixed methods approach for mapping water safety planning in Kenya. The premise of the manuscript is that it introduces a “participatory mapping” to rural water safety planning –a method that has never been applied to the empirical study or practice of rural water safety planning.

Generally speaking, this manuscript presents an interesting methodology and case study. It is well written and is relevant; however, it warrants some revisions to render it publishable in PLOS One. In order to be constructive, I structure my impressions of the article around two interrelated aspects: 1) the theoretical framing of the manuscript (i.e., the literature review); and 2) the empirical framing of the manuscript (i.e., research methods and analysis).


1) The theoretical framing of the manuscript (i.e., the literature review)

COMMENT: Basically, this component is missing from this manuscript. While the introduction is well written, it does not situate the manuscript’s topic “planning for rural water safety” within the larger body of debates on the subject matter. What is the status of rural water safety planning generally speaking, in Africa, and in Kenya particularly? what are the primary concerns with regards to water planning in rural Kenya? how has participatory planning for rural water safety achieved in general, in Africa, and in Kenya? and why is a participatory planning for rural water safety important (in comparison to other methods/approaches)? Including this discussion will render manuscript relevant to a larger audience of readers and will render some components of it transferable (e.g., the methods) and generalizable (e.g., the findings).

*RESPONSE: We accept that whilst we had explained water safety planning and the challenges for rural areas in our Introduction, we had not summarised its status and uptake in different settings. We now do so in a revised paragraph 1, drawing on relevant references including some helpfully suggested by the reviewer (one of which we had cited in our original manuscript though not in the Introduction). To strengthen the rationale for exploring participatory mapping in relation to rural water safety planning, we have broken paragraph 2 of our original Introduction out into two new paragraphs (now forming paragraphs 2 and 3 of the revised Introduction). Paragraph 2 sets out the challenges of implementing water safety planning in rural areas of developing countries. Via a deepened literature synthesis, Paragraph 3 reports on the ‘state of the art’, summarising efforts to simplify water safety planning in such settings, and participatory methods previously applied for engaging with community groups such as water user committees.*

COMMENT: Similarly, and because the manuscript is built on the claim that “participatory mapping” has never been applied to the empirical study or practice of rural water safety planning, the authors should introduce what exactly is “participatory mapping”? How has it been applied in the past, for which aspects of rural planning, and in which rural contexts within Africa and/or Kenya? And how is this method transferable to mapping rural water safety in particular? And, building on the previous question, why is this method optimal? Again, through addressing these questions, the readers would be provided with a context for this study.

Some references that should be consulted in this process include, but are not limited to:

I. On participatory mapping in Africa (note that some actually pertain to rural water planning):
a) Fagerholm, N., & Käyhkö, N. (2009). Participatory mapping and geographical patterns of the social landscape values of rural communities in Zanzibar, Tanzania. Fennia - International Journal of Geography, 187(1), 43-60. Retrieved from <https://fennia.journal.fi/article/view/3703>
b) Jiri Panek (2015) How participatory mapping can drive community empowerment – a case study of Koffiekraal, South Africa, South African Geographical Journal, 97:1, 18-30, DOI: 10.1080/03736245.2014.924866
c) Lammerink, Marc P. “Community Managed Rural Water Supply: Experiences from Participatory Action Research in Kenya, Cameroon, Nepal, Pakistan, Guatemala and Colombia.” Community Development Journal, vol. 33, no. 4, 1998, pp. 342–352. JSTOR, [www.jstor.org/stable/44258804](http://www.jstor.org/stable/44258804).

II. On participatory mapping in general and/or in contexts outside Africa:
d) Chambers, R. (2006), Participatory Mapping and Geographic Information Systems: Whose Map? Who is Empowered and Who Disempowered? Who Gains and Who Loses?. The Electronic Journal of Information Systems in Developing Countries, 25: 1-11. <https://doi.org/10.1002/j.1681-4835.2006.tb00163.x>
e) M. Anwar Hossen (2016) Participatory mapping for community empowerment, Asian Geographer, 33:2, 97-113, DOI: 10.1080/10225706.2016.1237370
f) C.E. Roa García, S. Brown (2009). Assessing water use and quality through youth participatory research in a rural Andean watershed, Journal of Environmental Management, Volume 90, Issue 10, Pages 3040-3047

*RESPONSE: We agree we should have more tightly defined the manuscript’s fit to prior knowledge via a more extensive and deeper literature review. We also thank the reviewer for taking the time to highlight specific relevant references for us. Paragraph 3 of the Introduction of our original manuscript provided a brief summary of participatory mapping and its applications. In the light of the reviewer’s comments and request that we deepen the literature review and more clearly explain the specific contribution of the manuscript, we have expanded this paragraph to incorporate some of the references above, strengthening the case for its application to rural water safety planning in doing so. We also clarify that we are not proposing that participatory mapping is the optimal method for rural water safety planning. Rather, we argue it is one of several relevant participatory techniques with potential for water safety planning. As such, we seek to provide evidence on its usefulness or otherwise through triangulation with other sources of information.*

2) The research methods and analyses:
This discussion that spreads over sections 2 and 3 is well-written and reflects a sound and strong methodology. However, it is extremely lengthy and overly detailed –to the extent that the bigger picture behind this empirical study is diluted. I recommend condensing these sections by at least a third each, if not more, and elaborating on the bigger picture and the relevance of the study’s findings to planning for rural water safety within Kenya specifically, but also, how does this study (whether its methods and/or findings) inform planning for rural water safety in Africa and beyond. In other words, the manuscript should highlight this study’s contribution and insights for rural Kenya and beyond.

*RESPONSE: As suggested, we have shortened Section 2 (Methods) by about a third for brevity, reducing methodological detail in doing so. To shorten Section 3 (Results), we have deleted Tables 1 and 4, summarising key statistics in the text instead. We also moved Figure 1 and Table 7 to Supplemental Materials, thereby nearly halving the number of illustrations. In reducing the number of illustrations, we have also shortened the accompanying text throughout for conciseness and removed a sub-section heading to simplify Section 3’s structure.*

## Reviewer #2:

COMMENT: This is a good study that empowers local communities to engage in water security. The ability to triangulate the outcomes between the surveys and the participatory mapping was critical to demonstrating the effectiveness of their methods.

RESPONSE: We thank the reviewer for these positive comments; no other response needed.

COMMENT: However, the overall article ends weakly. In particular, the last two sentences let the reader down. The next steps and recommendations for future research should be clearly articulated at the end of the conclusion section. Some of this is hinted at in the discussion, finish strongly.

*RESPONSE: Having revisited the literature on water safety planning and participatory mapping as suggested by Reviewer 1, we agree that we should have drawn out more in our Discussion and Conclusions. With this in mind, we have introduced a new sixth paragraph in the Discussion, where we reflect on the complexity of rural water source use and the value of informal and household-managed sources to the communities. We now highlight the application of rural water safety planning to such sources as a specific area for future research via an extended Conclusion.*
