# Supplementary material for: A mixed methods study to evaluate participatory mapping for rural water safety planning in western Kenya
Source: PLoS One. 2021 Jul 28;16(7):e0255286. doi: 10.1371/journal.pone.0255286 (PMC8318241; doi:10.1371/journal.pone.0255286)
Supplement: Attachment — Submitted filename: PONE-D-20-28371 Review_SW-2021-05-12.docx [file pone.0255286.s010.docx]

**Response to requested changes**

| **Comment** | **Lines (Based on the revised manuscript pdf)** | **Revision** | **Status** |
| --- | --- | --- | --- |
| Confusing sentence: “perceptions of what constitutes a contamination hazard consequent implications for hazard management | 75-76 | Perceptions of what constitutes a contamination hazard AND consequent implications for hazard management | **Incomplete**  Sentence was not changed. |
| Introduction does not situate manuscript’s topic “planning for rural water safety” | 61-62 | It was initially developed for urban utilities, but even in urban areas, its uptake has been slow globally | **Complete** |
| What is the status of rural water safety planning generally speaking, in Africa, and in Kenya particularly? | 62-64 | Generally, Sub-Saharan Africa has lagged behind  other world regions, with initial uptake in Uganda by 2011 (4), and a with subsequent more widespread uptake elsewhere in Africa including by Kenyan utilities (5). | **Complete**  More information on the status of rural water safety planning in Kenya specifically would help strengthen this |
| What are the primary concerns with regards to water planning in rural Kenya? | 68-70 | Further challenges exist in promoting uptake of water safety planning in rural areas. Particularly in developing countries, limited resources and remoteness make the dissemination and uptake of such procedures challenging | **Complete** |
| How has participatory planning for rural water safety achieved in general, in Africa, and in Kenya? | 85-98 | Responding to rural water safety planning challenges, WHO developed a simplified water safety planning workflow for small-scale supplies (2, 6), which involves six tasks: community engagement and team assembly; community supply description; identification of hazards and existing control measures; development and implementation of an improvement plan; monitoring of plan effectiveness; and documentation and review. A subsequent systematic review (9) highlighted the need for further simplification and community engagement in rural WSPs. | **Complete** |
| Why is a participatory planning for rural water safety important (in comparison to other methods/approaches)? |  | Participatory methods used in rural WSPs have included group hazard elicitation, ranking and scoring techniques (5, 13), role-playing (14), and educational games (13). Typically, WSP community engagement has focussed on formally managed supplies such as piped systems on boreholes, but not on household-managed sources such as wells or rainwater systems or informal sources such as surface water collection points. | **Complete**  Could further compare the different participatory methods and the results of them |
| Introduce what exactly is “participatory mapping”? | 96-98 | Participatory mapping has also been used to support community engagement during the early stages of water planning (13) and to support water resources management without a specific focus on water safety (15). | **Complete** |
| How has it been applied in the past, for which aspects of rural planning, and in which rural contexts within Africa and/or Kenya? | 104-107 | An early participatory mapping study (15) facilitated an action research partnership between Non-Governmental Organisations and local communities in Kenya and five other countries. Communities formed committees and began planning and mapping water resources through village walks. | **Complete** |
| How is this method transferable to mapping rural water safety in particular? | 112-113 | However, the approach has not yet been applied evaluated for to rural water safety planning (9) through triangulation with other information sources. | **Complete** |
| Why is this method optimal? |  |  | **Incomplete** |
| How does this study (whether its methods and/or findings) inform planning for rural water safety in Africa and beyond | 533-535 | Therefore, future research should also assess how far the rural water safety planning approach can be applied to informal and household-managed sources alongside the formal sources that have largely been the focus of water safety planning to date. | **Complete** |
| Condensing sections |  |  | **Complete** |
