# Supplementary material for: A mixed methods study to evaluate participatory mapping for rural water safety planning in western Kenya
Source: PLoS One. 2021 Jul 28;16(7):e0255286. doi: 10.1371/journal.pone.0255286 (PMC8318241; doi:10.1371/journal.pone.0255286)
Supplement: Attachment — Submitted filename: Okotto-Okottoetal_Response_ReviewersComments_June2021.docx [file pone.0255286.s011.docx]

# Response to Reviewers’ Comments: “A mixed methods study to evaluate participatory mapping for rural water safety planning in western Kenya” (Okotto-Okotto et al; Manuscript Number: PONE-D-20-28371)

Reviewer #1: 

We thank reviewer 1 for taking time to provide a very diligent and thorough review of our manuscript, which we also believe has greatly improved it. Responses to the remaining outstanding comments are provided below.

*COMMENT 1) Confusing sentence: “perceptions of what constitutes a contamination hazard consequent implications for hazard management 75-76 Sentence was not changed.*

RESPONSE: We have reworded this sentence to make our meaning clearer (paragraph 2 of Introduction).

*COMMENT: 2) What is the status of rural water safety planning generally speaking, in Africa, and in Kenya particularly? 62-64 Generally, Sub-Saharan Africa has lagged behind other world regions, with initial uptake in Uganda by 2011 (4), and a with subsequent more widespread uptake elsewhere in Africa including by Kenyan utilities (5). More information on the status of rural water safety planning in Kenya specifically would help strengthen this.*

RESPONSE: One of the reasons why we struggled to respond to this component of the reviewer’s suggestion in our previous submission is that there is actually minimal published evidence on rural water safety planning in Kenya. However, in our revised version, we now summarise how far the latest government guidance in Kenyan covers the situation in rural areas (final sentences of paragraph 3 in our revised Introduction).

*COMMENT: 3) Why is this method optimal? Incomplete*

RESPONSE: We now briefly summarise the reasons as to why participatory mapping is very well suited to water safety planning, drawing on WHO recommendations advocating participatory mapping for water safety planning of small community supplies in doing so (Introduction, penultimate paragraph).
